# Inflammation, cell death, and lncRNAs: unraveling the mechanisms of sepsis-associated acute kidney injury

**DOI:** 10.3389/fimmu.2026.1710624

**Published:** 2026-03-03

**Authors:** Dongsheng Ren, Qinghai Liu, Haitang Liao, Ming Wang, Yiyan Wang, Wenhui Guo, Chenyang Duan, Yuling Zhou, Zhenchun Luo

**Affiliations:** 1 Department of Critical Care Medicine, Chongqing Hospital of Traditional Chinese Medicine, Chongqing, China; 2 Department of Cardiology, Chongqing Hospital of Traditional Chinese Medicine, Chongqing, China; 3 Department of Anesthesiology, The Second Affiliated Hospital of Chongqing Medical University, Chongqing, China; 4 Basic Medicine Research and Innovation Center for Novel Target and Therapeutic Intervention, Ministry of Education, the Second Affiliated Hospital of Chongqing Medical University, Chongqing, China

**Keywords:** biomarkers and therapeutic targets, inflammation and oxidative stress, long non-coding RNAs, programmed cell death, sepsis-associated acute kidney injury

## Abstract

Sepsis-associated acute kidney injury (SA-AKI) is a common and devastating complication of sepsis and remains a major contributor to morbidity and mortality in critically ill patients. Despite advances in supportive care, effective pharmacological therapies are still lacking, largely due to the complex and multifactorial pathogenesis of SA-AKI. Accumulating evidence indicates that dysregulated inflammation, oxidative stress, and multiple forms of programmed cell death—including apoptosis, pyroptosis, ferroptosis, and autophagy—are central drivers of renal tubular and endothelial dysfunction during sepsis. Recent studies have identified long non-coding RNAs (lncRNAs) as critical regulators of these pathogenic processes. Through competing endogenous RNA networks or direct interactions with proteins, lncRNAs modulate inflammatory signaling, oxidative stress responses, and cell fate decisions. This review summarizes current mechanistic insights into lncRNA-mediated regulatory networks in SA-AKI, highlights representative molecular axes defined in experimental models, and discusses the translational potential of lncRNAs as diagnostic biomarkers or therapeutic targets. Importantly, lncRNAs exhibit a context-dependent duality, acting as either pathogenic amplifiers or protective modulators of renal injury, underscoring both their biological complexity and clinical relevance in SA-AKI.

## 1 Introduction

Sepsis remains a leading cause of morbidity and mortality in intensive care units worldwide (1). Among its complications, sepsis-associated acute kidney injury (SA-AKI) represents one of the most severe and frequent organ dysfunctions, affecting up to 40–60% of critically ill patients and markedly increasing the risk of death (2, 3). Epidemiological studies consistently demonstrate that SA-AKI not only worsens short-term outcomes but also predisposes survivors to chronic kidney disease and long-term renal impairment (4, 5). Despite improvements in supportive management, such as hemodynamic stabilization, timely antibiotic therapy, and continuous renal replacement therapy (CRRT), there are still no specific pharmacological interventions proven to effectively prevent or reverse SA-AKI (6, 7). This underscores an urgent need to explore novel molecular mechanisms and therapeutic targets.

In recent years, non-coding RNAs have attracted growing attention as essential regulators of gene expression beyond traditional protein-coding paradigms (8, 9). Current genomic studies have revealed that protein-coding sequences account for less than 2% of the human genome, while more than 75% is pervasively transcribed into non-coding RNAs, highlighting the extensive regulatory potential of the non-coding transcriptome (10, 11). Among these, long non-coding RNAs (lncRNAs), defined as transcripts longer than 200 nucleotides without apparent protein-coding capacity, have emerged as highly versatile modulators of cellular signaling and stress responses (12, 13). Unlike microRNAs, which typically act through post-transcriptional silencing, lncRNAs exhibit remarkable mechanistic diversity. They can act as competing endogenous RNAs (ceRNAs) by sponging microRNAs and thereby regulating downstream mRNA targets, form functional scaffolds or decoys for RNA-binding proteins, influence chromatin remodeling and transcription factor recruitment in the nucleus, or modulate mRNA splicing, stability, and translation in the cytoplasm (14, 15). This multilayered regulatory capacity positions lncRNAs at the crossroads of transcriptional, post-transcriptional, and epigenetic networks. Experimental evidence increasingly demonstrates that dysregulated lncRNA expression profoundly influences the pathophysiology of sepsis-associated acute kidney injury (SA-AKI) (16, 17). On the pathogenic side, certain lncRNAs promote renal tubular damage by amplifying inflammatory signaling, facilitating NLRP3 inflammasome activation, or enhancing multiple forms of programmed cell death such as apoptosis, pyroptosis, and ferroptosis (18). For example, lncRNAs like PVT1 and MEG3 act through specific miRNA–mRNA axes to intensify NF-κB activation, gasdermin D–mediated pyroptosis, or oxidative stress, thereby worsening kidney dysfunction (18–20). Conversely, protective lncRNAs can mitigate renal injury by buffering excessive inflammation, suppressing oxidative damage, or activating pro-survival pathways. Molecules such as CASC2, GAS5, and XIST have been shown to restore mitochondrial homeostasis, attenuate NF-κB activity, or reduce apoptosis, ultimately conferring resistance to septic insults (21–23). Importantly, these divergent functions highlight the context-dependent and cell type–specific nature of lncRNA biology, suggesting that they function as dynamic molecular switches that fine-tune renal outcomes under septic stress.

Given these dual and context-dependent roles, lncRNAs are increasingly viewed as both mechanistic drivers and potential biomarkers or therapeutic targets in SA-AKI. The present review aims to provide a comprehensive overview of current knowledge on lncRNA-mediated regulatory mechanisms in SA-AKI, highlight their involvement across key pathogenic processes, and discuss opportunities and challenges for translating these findings into clinical practice.

## 2 Pathophysiological landscape of septic acute kidney injury

SA-AKI arises from multifactorial and interwoven pathogenic mechanisms that extend far beyond simple hemodynamic alterations such as hypoperfusion (24–26). A hallmark feature is dysregulated immunity, where pathogen-associated molecular patterns (PAMPs) like lipopolysaccharide (LPS) and damage-associated molecular patterns (DAMPs) released from injured tissues activate Toll-like receptors (TLRs) and other pattern recognition receptors on renal tubular and immune cells (27, 28). This initiates downstream NF-κB signaling, resulting in a robust surge of pro-inflammatory cytokines and chemokines including TNF-α, IL-1β, and IL-6 (29, 30). In parallel, activation of the NLRP3 inflammasome amplifies renal injury through caspase-1–mediated cleavage of pro-IL-1β and pro-IL-18, driving pyroptotic cell death and propagating inflammation in a feed-forward loop (31, 32). Oxidative stress further compounds the inflammatory burden. Excessive generation of reactive oxygen species (ROS) overwhelms endogenous antioxidant defenses, disrupts mitochondrial homeostasis, and impairs oxidative phosphorylation (33). Key redox-sensitive mediators such as thioredoxin-interacting protein (TXNIP) act as molecular links between oxidative stress and inflammasome activation, while mitochondrial dysfunction exacerbates energy deficits in metabolically demanding tubular epithelial cells. This redox imbalance also contributes to lipid peroxidation, setting the stage for ferroptosis. Multiple forms of programmed cell death converge to drive renal structural and functional deterioration (34). Classical apoptosis proceeds through mitochondrial cytochrome c release and caspase activation, leading to loss of tubular epithelial cells. Pyroptosis, mediated by inflammasome–caspase-1–gasdermin D signaling, induces cell swelling, pore formation, and release of pro-inflammatory mediators (35). Ferroptosis, a recently characterized form of regulated necrosis, is distinguished by iron-dependent lipid peroxidation and glutathione peroxidase 4 (GPX4) inactivation, linking metabolic reprogramming to cell death (36, 37). Autophagy adds another layer of complexity: moderate activation may promote cell survival by clearing damaged organelles and limiting oxidative injury, but excessive or dysregulated autophagy can aggravate tubular dysfunction, underscoring its dualistic role (38, 39). Beyond epithelial damage, endothelial injury and microvascular dysfunction are critical in SA-AKI pathogenesis. Endothelial activation leads to increased vascular permeability, glycocalyx degradation, and leukocyte adhesion, fostering microthrombosis and regional hypoxia. These alterations impair peritubular microcirculation and oxygen delivery, thereby synergizing with inflammation and metabolic stress to accelerate renal dysfunction (40, 41). Cross-talk between endothelial cells, immune cells, and tubular epithelium creates a vicious cycle that perpetuates tissue injury. Collectively, these processes create a hostile intrarenal milieu characterized by inflammatory amplification, oxidative imbalance, metabolic stress, and cell death, culminating in acute functional loss (42, 43). Importantly, emerging evidence demonstrates that long non-coding RNAs (lncRNAs) intersect with each of these pathways. By acting as competing endogenous RNAs, scaffolds for protein complexes, or regulators of transcriptional and post-transcriptional networks, lncRNAs can either exacerbate these injurious cascades or provide protective buffering (44, 45). Some lncRNAs drive NF-κB activation, inflammasome signaling, and ferroptosis, while others enhance antioxidant defenses, stabilize mitochondrial metabolism, or suppress apoptosis and pyroptosis (20, 22, 46–49). Thus, lncRNAs function as molecular switches that fine-tune the trajectory of renal injury in sepsis, positioning them as pivotal regulators of disease progression and highly attractive targets for biomarker discovery and therapeutic innovation in SA-AKI.

## 3 lncRNAs as regulators of key mechanisms in SA-AKI

Accumulating studies demonstrate that long non-coding RNAs (lncRNAs) exert profound effects on the molecular pathways underpinning SA-AKI. Acting primarily through ceRNA networks or direct interactions with proteins, lncRNAs can modulate inflammation, oxidative stress, cell death, and survival signaling in renal tubular and endothelial cells.

### 3.1 Inflammation and NF-κB signaling

**Figure 1:**
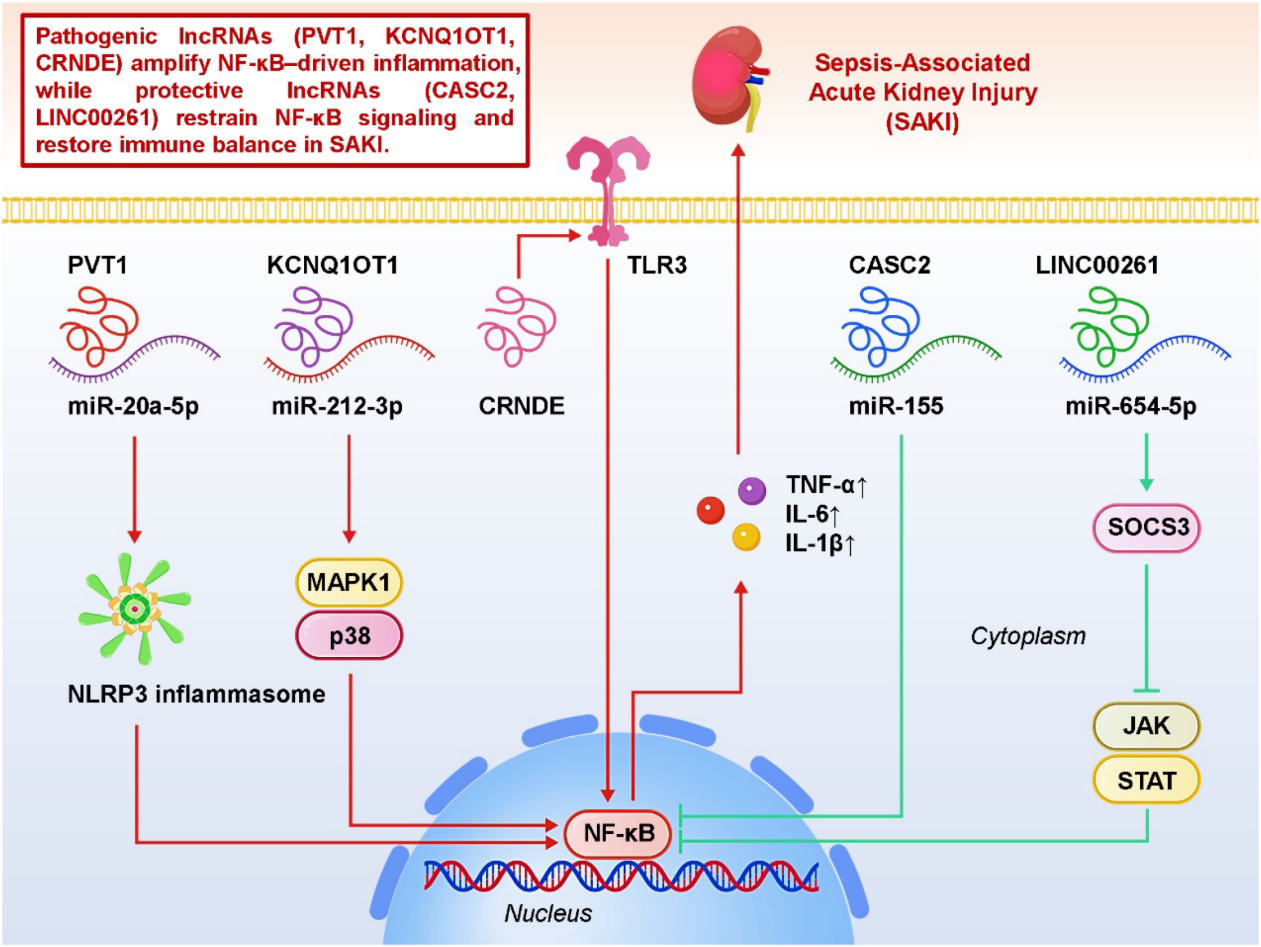
Regulatory roles of long non-coding RNAs (lncRNAs) in nuclear factor kappa B (NF-κB) signaling during sepsis-associated acute kidney injury (SA-AKI).

Building on the inflammatory signaling framework described in Section 3, long non-coding RNAs (lncRNAs) have emerged as critical upstream regulators of NF-κB activation in SA-AKI. Rather than initiating inflammatory cascades *de novo*, lncRNAs primarily fine-tune the magnitude and persistence of NF-κB signaling by modulating pathway priming, signal amplification, and negative feedback control. Through competing endogenous RNA (ceRNA) mechanisms or direct protein interactions, lncRNAs influence the transcription of pro-inflammatory cytokines and inflammasome-related genes, thereby shaping renal inflammatory outcomes under septic stress (50, 51). On the pathogenic side, PVT1 augments NF-κB activation and primes the NLRP3 inflammasome via the miR-20a-5p/NLRP3 axis, thereby amplifying transcription of pro-inflammatory cytokines and creating a feed-forward loop that exacerbates renal tubular injury; it also links to oxidative stress pathways, suggesting a dual role in both immune activation and redox imbalance (18). KCNQ1OT1, through its regulation of the miR-212-3p/MAPK1 axis, reinforces p38-mediated NF-κB signaling, further promoting the release of TNF-α and IL-6 and aggravating structural damage (47). Likewise, CRNDE has been shown to activate the TLR3/NF-κB pathway, underscoring the contribution of endosomal PRRs to renal inflammation and highlighting that lncRNAs can interface with distinct pathogen-sensing platforms (52). In contrast, protective lncRNAs counterbalance these effects: CASC2 acts as a sponge for miR-155, a microRNA well known to enhance NF-κB activation, thus limiting downstream cytokine transcription and improving renal outcomes; in addition, it may intersect with PPAR-related metabolic regulation, illustrating crosstalk between immunometabolism and inflammation (53). LINC00261 further demonstrates anti-inflammatory capacity by modulating the miR-654-5p/SOCS3 axis, strengthening negative feedback on JAK/STAT pathways that converge on NF-κB activity (54). Collectively, these studies reveal that lncRNAs can function as bidirectional modulators of renal inflammation—either acting as amplifiers that perpetuate NF-κB-driven injury or as brakes that restore immune homeostasis—thereby positioning them as promising mechanistic nodes for therapeutic intervention in SA-AKI. **Figure 1** illustrates how different lncRNAs positively or negatively regulate the NF-κB signaling pathway in SA-AKI, thereby either promoting inflammation or suppressing it, ultimately determining tubular epithelial cell injury or protection outcomes.

### 3.2 Pyroptosis and inflammasome activation

**Figure 2:**
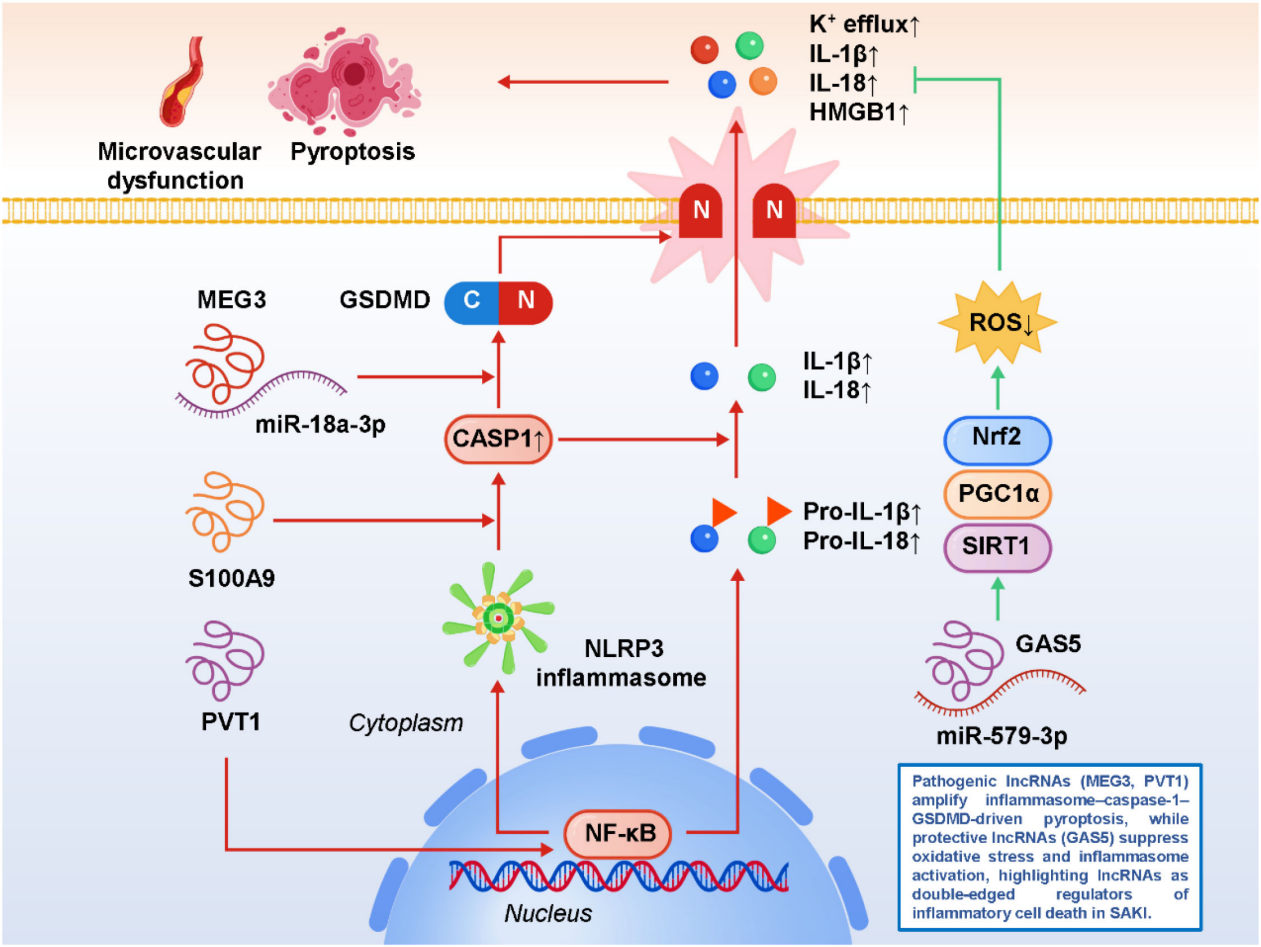
LncRNA-mediated regulation of pyroptosis, an inflammasome-dependent form of programmed cell death, in sepsis-associated acute kidney injury (SA-AKI).

As summarized in Section 3, pyroptosis represents a key inflammatory form of programmed cell death in SA-AKI, driven by NF-κB–dependent priming and subsequent activation of the NLRP3 inflammasome. Within this established framework, accumulating evidence indicates that lncRNAs act as pivotal modulators that control inflammasome assembly, caspase-1 activation, and execution of pyroptotic cell death in renal tubular epithelial cells. For example, MEG3 facilitates pyroptotic cell death through the miR-18a-3p/GSDMD pathway, relieving repression of GSDMD and promoting its cleavage by caspase-1 into the pore-forming N-terminal fragment (20). This enhances membrane permeabilization, potassium efflux, and downstream inflammasome activation, which together aggravate tubular damage. PVT1 further reinforces this process by driving NLRP3 inflammasome activation, integrating NF-κB-dependent priming (increasing NLRP3 and pro-IL-1β transcription) with inflammasome assembly and caspase-1 cleavage (55). In doing so, PVT1 effectively bridges transcriptional activation of pro-inflammatory genes with the execution of pyroptosis, serving as a central amplifier of inflammatory cell death in sepsis. In addition to lncRNAs, inflammatory mediators such as S100A9 have been implicated in the amplification of pyroptotic injury by enhancing caspase-1 activity and modulating inflammasome dynamics, suggesting that lncRNAs may act in synergy with classical DAMPs to potentiate renal inflammation (56). Conversely, protective lncRNAs play an important counter-regulatory role. For example, GAS5 exerts an anti-pyroptotic effect by repressing miR-579-3p, thereby activating the SIRT1/PGC-1α/Nrf2 axis. This enhances antioxidant defenses, restores mitochondrial homeostasis, and reduces ROS-mediated inflammasome activation, ultimately attenuating pyroptotic death and preserving tubular epithelial integrity (22). Other protective candidates, though less studied, are likely to exist and may contribute to maintaining immune balance and limiting excessive cell lysis. These findings highlight pyroptosis as a critical intersection point where pathogenic lncRNAs amplify renal injury while protective lncRNAs confer resilience, underscoring the double-edged role of non-coding RNA networks. Importantly, they also raise translational opportunities. Inhibiting pathogenic lncRNAs such as MEG3 or PVT1—through antisense oligonucleotides, siRNAs, or small-molecule modulators—may suppress excessive inflammasome activation, whereas enhancing protective transcripts like GAS5 could bolster intrinsic defense mechanisms (57, 58). Furthermore, pharmacological agents that indirectly modulate pyroptosis-related lncRNA pathways, including resveratrol or curcumin, may serve as adjunctive strategies in sepsis therapy. However, translating these insights into clinical interventions requires careful consideration of timing and context, as complete suppression of inflammasome activity could compromise host defense. Thus, future research should aim to delineate cell type–specific roles of lncRNAs, validate their functions in large-animal and clinical cohorts, and develop kidney-targeted delivery systems to selectively modulate pyroptosis-related networks. In summary, lncRNA-mediated regulation of pyroptosis exemplifies the intricate interplay between innate immune activation, programmed cell death, and renal dysfunction in sepsis. By selectively targeting lncRNA–miRNA–inflammasome axes, it may be possible to attenuate the excessive inflammatory cell death that drives SA-AKI, paving the way for novel, mechanism-based therapies that complement existing supportive care. **Figure 2** illustrates how pathogenic and protective lncRNAs differentially regulate inflammasome–caspase-1–GSDMD signaling and pyroptosis in SA-AKI, shaping the balance between tubular injury and cellular protection.

### 3.3 Ferroptosis and oxidative stress

**Figure 3:**
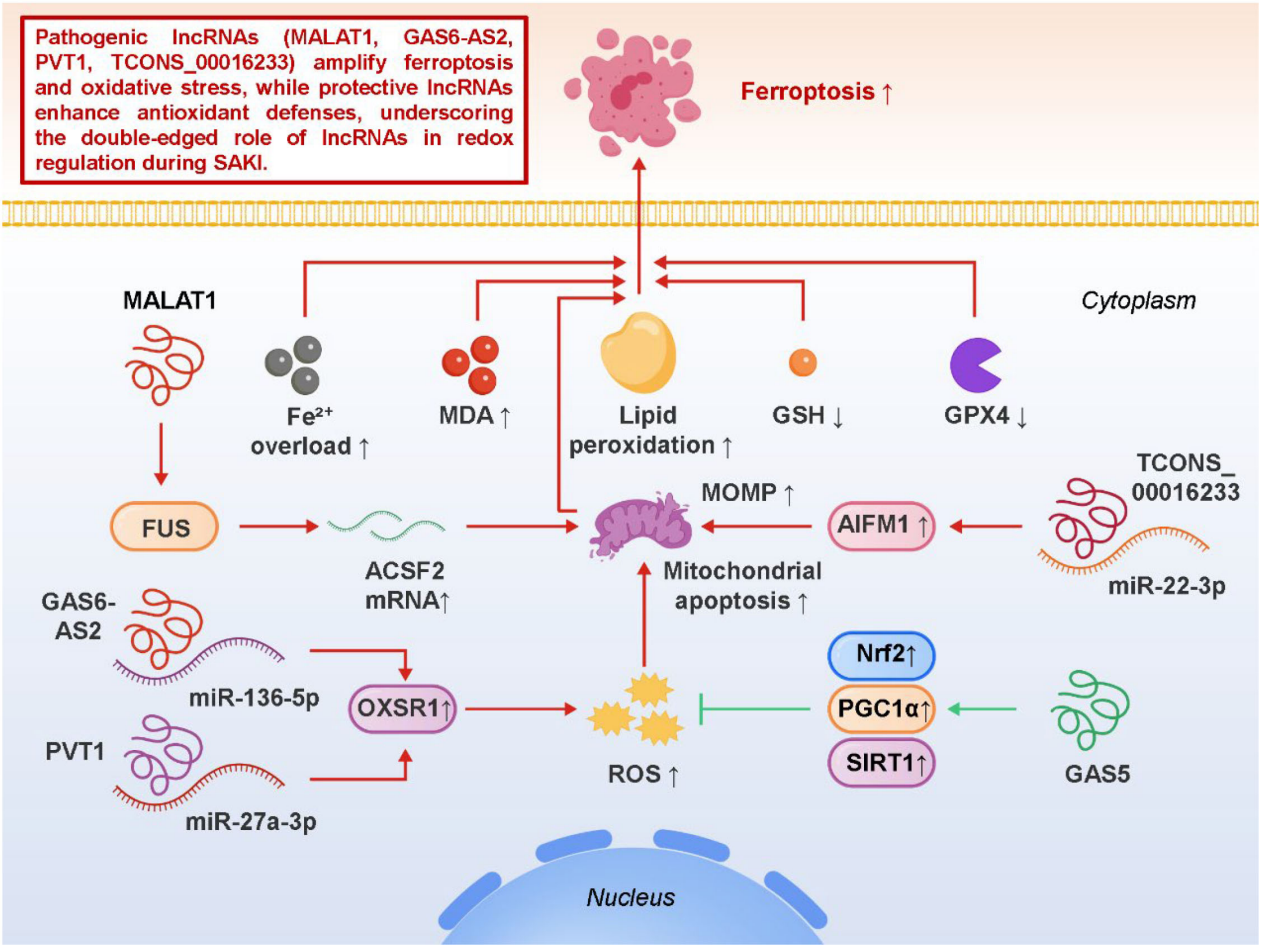
LncRNA-mediated regulation of ferroptosis and oxidative stress in sepsis-associated acute kidney injury (SA-AKI).

Within the oxidative stress–mitochondrial dysfunction axis outlined in Section 3, ferroptosis has emerged as an important contributor to renal tubular injury during sepsis. Rather than reiterating the core biochemical features of ferroptosis, recent studies emphasize that lncRNAs modulate ferroptotic susceptibility by regulating lipid peroxidation, iron homeostasis, and antioxidant defense pathways, thereby linking redox imbalance to tubular cell fate decisions in SA-AKI. Accumulating evidence has identified lncRNAs as important regulators of ferroptotic signaling. MALAT1, one of the most studied lncRNAs, promotes ferroptosis by interacting with the RNA-binding protein FUS, which stabilizes ACSF2 mRNA and enhances lipid metabolic flux, thereby facilitating the production of peroxidation-prone substrates. In SA-AKI models, MALAT1 upregulation correlates with increased lipid ROS, suppressed GPX4 activity, and worsened tubular damage (59). Beyond MALAT1, other lncRNA–miRNA–mRNA axes also contribute to oxidative injury. GAS6-AS2, by sponging miR-136-5p and upregulating OXSR1, drives excessive ROS generation and disrupts antioxidant defenses, while PVT1, through the miR-27a-3p/OXSR1 axis, reinforces similar redox imbalance, underscoring its role as a multifaceted amplifier of septic pathology (60, 61). These findings highlight the convergence of different lncRNAs on common oxidative stress mediators, suggesting functional redundancy and robustness in the pathogenic network. Importantly, oxidative stress is not an isolated pathway but closely interfaces with mitochondrial dysfunction. ROS accumulation impairs mitochondrial membrane potential, disrupts ATP production, and triggers mitochondrial outer membrane permeabilization (MOMP) (62–64). For example, TCONS_00016233 regulates the miR-22-3p/AIFM1 axis, which enhances mitochondrial permeability transition and promotes caspase-dependent apoptosis, illustrating how oxidative stress and ferroptosis cross-talk with classical cell death programs. This integration means that a single lncRNA-driven alteration in redox balance can tilt the cell toward multiple death modalities, amplifying renal injury in sepsis (65). From a translational standpoint, these insights place lncRNAs at the center of ferroptosis and oxidative stress regulation, offering opportunities for therapeutic targeting. Silencing pathogenic lncRNAs such as MALAT1, GAS6-AS2, or PVT1 could limit ROS accumulation, preserve GPX4 activity, and reduce ferroptotic damage, while enhancing antioxidant pathways through protective lncRNAs may further bolster cellular resilience. Pharmacological modulators of ferroptosis, including iron chelators, lipophilic antioxidants, and GPX4 stabilizers, could potentially be combined with lncRNA-targeted interventions to achieve synergistic protection. However, challenges remain, including the need to validate ferroptosis-specific biomarkers in patients, to dissect cell type–specific lncRNA functions, and to develop kidney-targeted RNA delivery systems. Collectively, current evidence highlights ferroptosis as a critical and underexplored contributor to SA-AKI, with lncRNAs acting as master regulators of redox balance and lipid peroxidation (66–68). By modulating lncRNA–miRNA–redox axes, it may be possible to mitigate oxidative damage, prevent the collapse of mitochondrial homeostasis, and preserve renal tubular integrity during sepsis, thereby opening a promising frontier for both mechanistic research and therapeutic development. **Figure 3** illustrates how pathogenic and protective lncRNAs orchestrate ferroptosis and oxidative stress in SA-AKI, highlighting their dual roles in driving injury or preserving renal tubular cell survival.

### 3.4 Autophagy regulation

**Figure 4:**
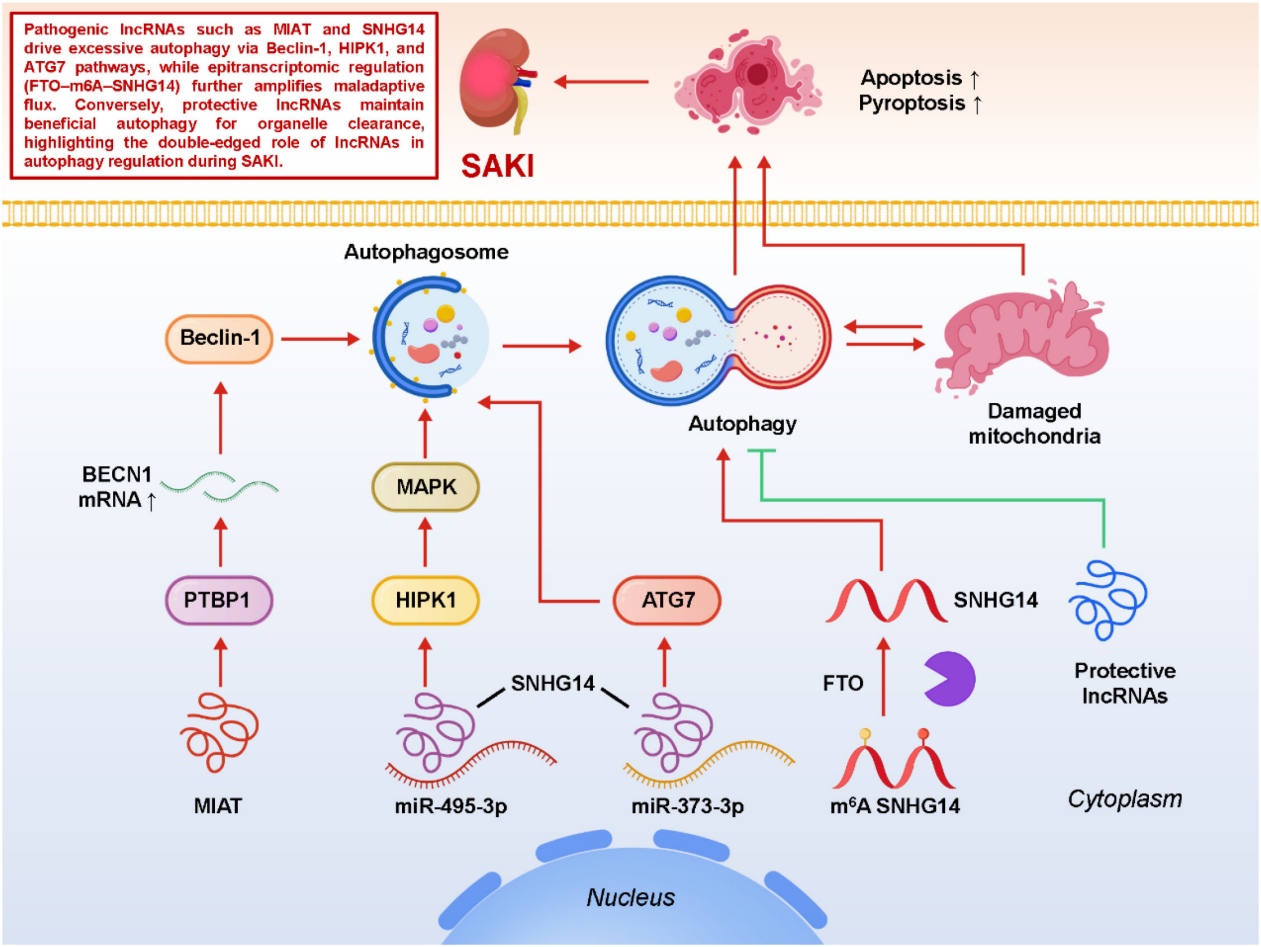
LncRNA-mediated regulation of autophagy, a lysosome-dependent degradative pathway, in sepsis-associated acute kidney injury (SA-AKI).

Autophagy, a highly conserved lysosomal degradation pathway that clears damaged proteins, lipids, and organelles, plays a particularly complex role in the setting of SA-AKI (69, 70). In physiological states, basal autophagy maintains renal tubular homeostasis by ensuring mitochondrial quality control, balancing energy metabolism, and preventing accumulation of misfolded proteins. Under septic stress, however, autophagy can act as a double-edged sword: moderate activation is generally adaptive, helping cells cope with oxidative and inflammatory insults, whereas excessive or dysregulated autophagy may consume essential cellular components, aggravate energy deficits, and ultimately contribute to tubular epithelial dysfunction (71–73). This context dependence makes autophagy a finely tuned process, and recent evidence suggests that lncRNAs are central regulators orchestrating its magnitude, timing, and outcome. Among the lncRNAs implicated, MIAT has been shown to interact with the RNA-binding protein PTBP1, thereby stabilizing BECN1 mRNA and enhancing Beclin-1–dependent autophagy. This promotes autophagosome formation and lysosomal flux, providing an initial buffer against overwhelming stress in septic tubular cells (74). However, sustained MIAT overexpression can tip the balance toward maladaptive autophagy, highlighting the importance of temporal dynamics in lncRNA-mediated regulation. SNHG14 represents another multifaceted regulator: through the miR-495-3p/HIPK1 axis, it influences MAPK-mediated stress signaling, which indirectly shapes autophagic responses; and via the miR-373-3p/ATG7 pathway, it directly governs autophagosome initiation, positioning SNHG14 as a nodal hub coupling inflammatory stimuli with autophagy machinery (75). This dual regulation underscores how a single lncRNA can integrate multiple signaling pathways to fine-tune cellular fate. Beyond canonical ceRNA interactions, epitranscriptomic regulation adds an additional layer of complexity. For instance, the RNA demethylase FTO mediates m6A demethylation of SNHG14, which stabilizes its transcript and alters autophagic flux. This demonstrates that RNA modifications can indirectly reshape renal stress responses by modulating lncRNA abundance and function (76). Such findings expand the scope of autophagy regulation from transcriptional and post-transcriptional levels to the epitranscriptomic landscape, suggesting potential synergies between RNA modification enzymes and lncRNAs in controlling SA-AKI outcomes. Mechanistically, lncRNA-dependent modulation of autophagy intersects with other pathogenic processes in SA-AKI. ROS accumulation and mitochondrial dysfunction, for example, serve as both triggers and targets of lncRNA-regulated autophagy, creating feedback loops that can either restore cellular equilibrium or intensify injury. Likewise, excessive autophagy may converge with apoptosis or pyroptosis, blurring the boundaries between distinct death programs and amplifying renal damage. This crosstalk suggests that therapeutic modulation of autophagy-related lncRNAs should be approached with precision, taking into account not only the extent of autophagy but also its interaction with parallel injury pathways. From a translational perspective, targeting lncRNAs that govern autophagy offers both promise and challenge (77, 78). Inhibiting maladaptive lncRNAs such as MIAT or SNHG14 may help restrain excessive autophagic flux, while enhancing protective lncRNAs could promote beneficial organelle clearance and cytoprotective adaptation. Advances in RNA-based therapeutics, including antisense oligonucleotides, siRNAs, and CRISPR-mediated interventions, provide the technical means to achieve such modulation (79–82). Yet, several hurdles remain: the context dependence of autophagy necessitates careful timing of intervention, kidney-specific delivery platforms must be optimized to avoid systemic side effects, and large-animal or clinical validation is essential to ensure that findings from rodent models can be extrapolated to human sepsis. In sum, lncRNA-mediated control of autophagy in SA-AKI is highly dynamic and context-specific, with outcomes ranging from protective to deleterious. By dissecting the molecular networks through which lncRNAs influence autophagic pathways—and by developing strategies to selectively harness beneficial autophagy while avoiding its pathological overactivation—future studies may open new therapeutic avenues to mitigate septic renal injury. **Figure 4** illustrates how lncRNAs orchestrate maladaptive versus protective autophagy in SA-AKI, integrating transcriptional, epitranscriptomic, and stress-responsive pathways to determine renal tubular fate.

### 3.5 Apoptosis and cellular survival pathways

**Figure 5:**
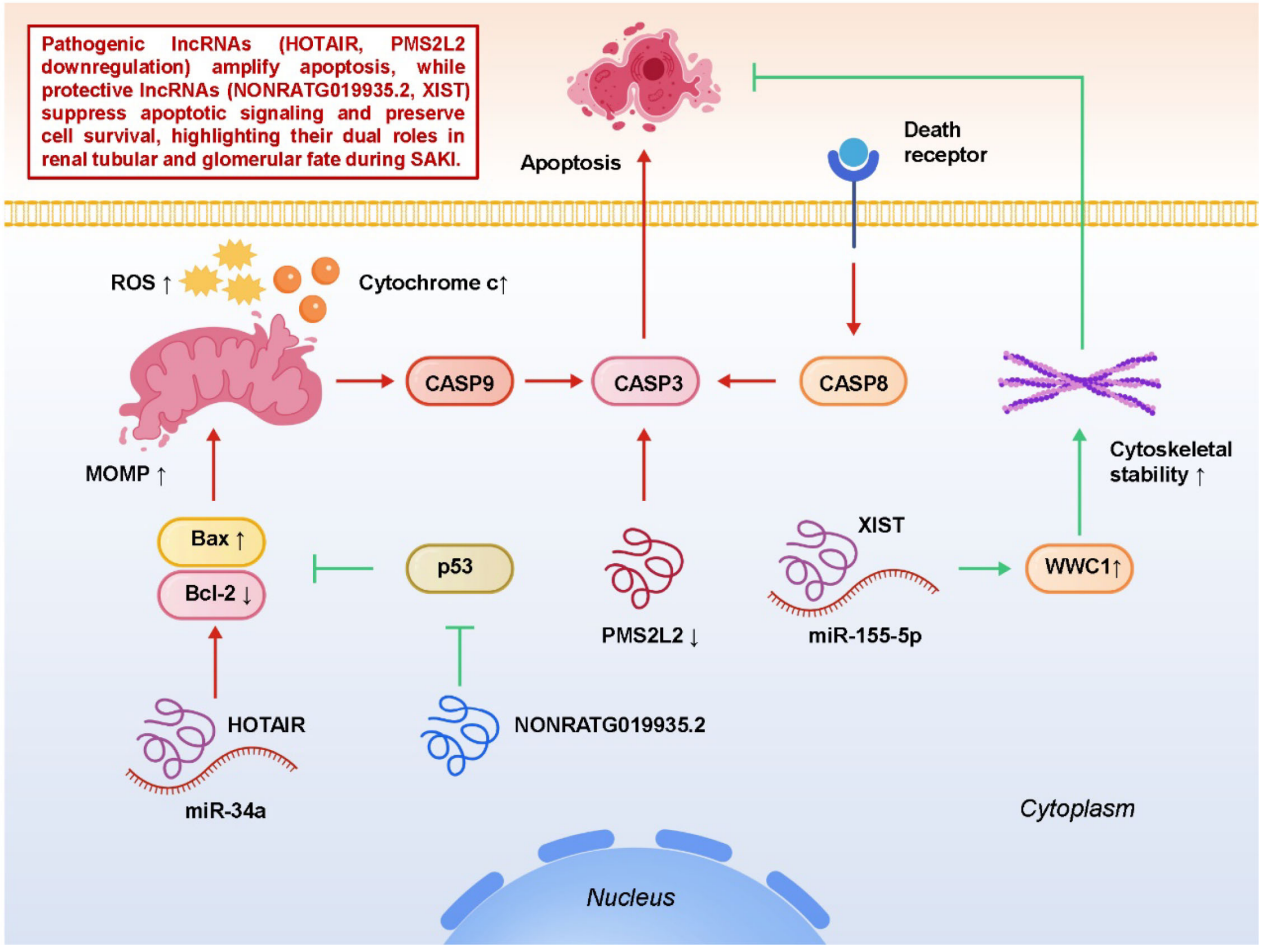
LncRNA-mediated regulation of apoptosis and cell survival signaling pathways in sepsis-associated acute kidney injury (SA-AKI).

In the context of mitochondrial dysfunction and oxidative stress described in Section 3, lncRNAs further modulate apoptotic signaling pathways in sepsis-associated acute kidney injury. By influencing the balance between pro-apoptotic and anti-apoptotic regulators, lncRNAs integrate inflammatory and metabolic stress cues to determine tubular and glomerular cell survival under septic conditions. LncRNAs, through ceRNA activity or direct protein interactions, fine-tune the balance between pro-apoptotic and anti-apoptotic mediators, thereby determining renal cell fate under septic conditions. On the protective side, NONRATG019935.2 has been shown to attenuate tubular cell apoptosis by suppressing p53-mediated transcriptional programs, thereby limiting the upregulation of pro-apoptotic genes such as Bax and PUMA (83). By dampening mitochondrial pore formation and cytochrome c release, this lncRNA preserves cellular viability during septic stress and highlights how non-coding transcripts can constrain canonical tumor suppressor pathways in a beneficial context. Similarly, XIST exerts renoprotective effects by modulating the miR-155-5p/WWC1 axis, which stabilizes cytoskeletal architecture, supports cell–cell junction integrity, and suppresses downstream caspase activation (23). These findings suggest that protective lncRNAs not only reduce apoptotic signaling but also maintain structural resilience in tubular epithelial cells. In contrast, several lncRNAs function as pro-apoptotic drivers. HOTAIR, through the miR-34a/Bcl-2 axis, represses the anti-apoptotic protein Bcl-2, tipping the balance toward Bax-mediated mitochondrial permeabilization, cytochrome c release, and activation of caspase-3, thereby intensifying renal tubular injury (84). Reduced expression of PMS2L2 has also been linked to enhanced podocyte apoptosis, implicating lncRNAs in glomerular cell fate decisions that compromise barrier integrity and contribute to proteinuria in septic contexts (85). These observations extend the influence of lncRNAs beyond tubular compartments, suggesting a role in endothelial and glomerular injury as well. Mechanistically, lncRNA-mediated apoptotic regulation often overlaps with other cell death programs, such as pyroptosis and ferroptosis, reflecting the interconnected nature of regulated cell death in SA-AKI. For instance, mitochondrial ROS accumulation not only triggers apoptosis but also primes inflammasome activation and lipid peroxidation, meaning that lncRNA-driven redox imbalance can shift the cell toward multiple modes of demise (86–88). This underscores the notion that lncRNAs act as molecular integrators, channeling diverse stress signals into a final decision on survival versus death. From a translational perspective, the dual roles of lncRNAs in apoptosis underscore their therapeutic potential. Selective silencing of pro-apoptotic lncRNAs (e.g., HOTAIR) using antisense oligonucleotides or siRNAs could restrain excessive tubular apoptosis, while augmentation of protective transcripts (e.g., NONRATG019935.2 or XIST) through lncRNA mimics or gene therapy might bolster renal resilience. Moreover, circulating or urinary levels of apoptosis-related lncRNAs may serve as non-invasive biomarkers for early detection of tubular injury or for monitoring therapeutic responses in sepsis. However, careful validation across multiple models and patient cohorts is needed, given the cell type– and context-specific variability observed in lncRNA function. In summary, lncRNAs function as both accelerators and inhibitors of apoptotic signaling in SA-AKI, influencing not only tubular epithelial cells but also podocytes and endothelial compartments. By fine-tuning the balance between pro- and anti-apoptotic regulators, lncRNAs exert a decisive influence on renal outcomes in sepsis. Targeting these transcripts holds considerable promise for preserving renal cell survival, improving functional recovery, and ultimately enhancing prognosis in critically ill patients. **Figure 5** depicts how pathogenic and protective lncRNAs orchestrate mitochondrial apoptosis and survival pathways in SA-AKI, shaping renal tubular and glomerular fate through a balance of pro-death and pro-survival signals.

### 3.6 Contradictory findings and context dependence

**Figure 6:**
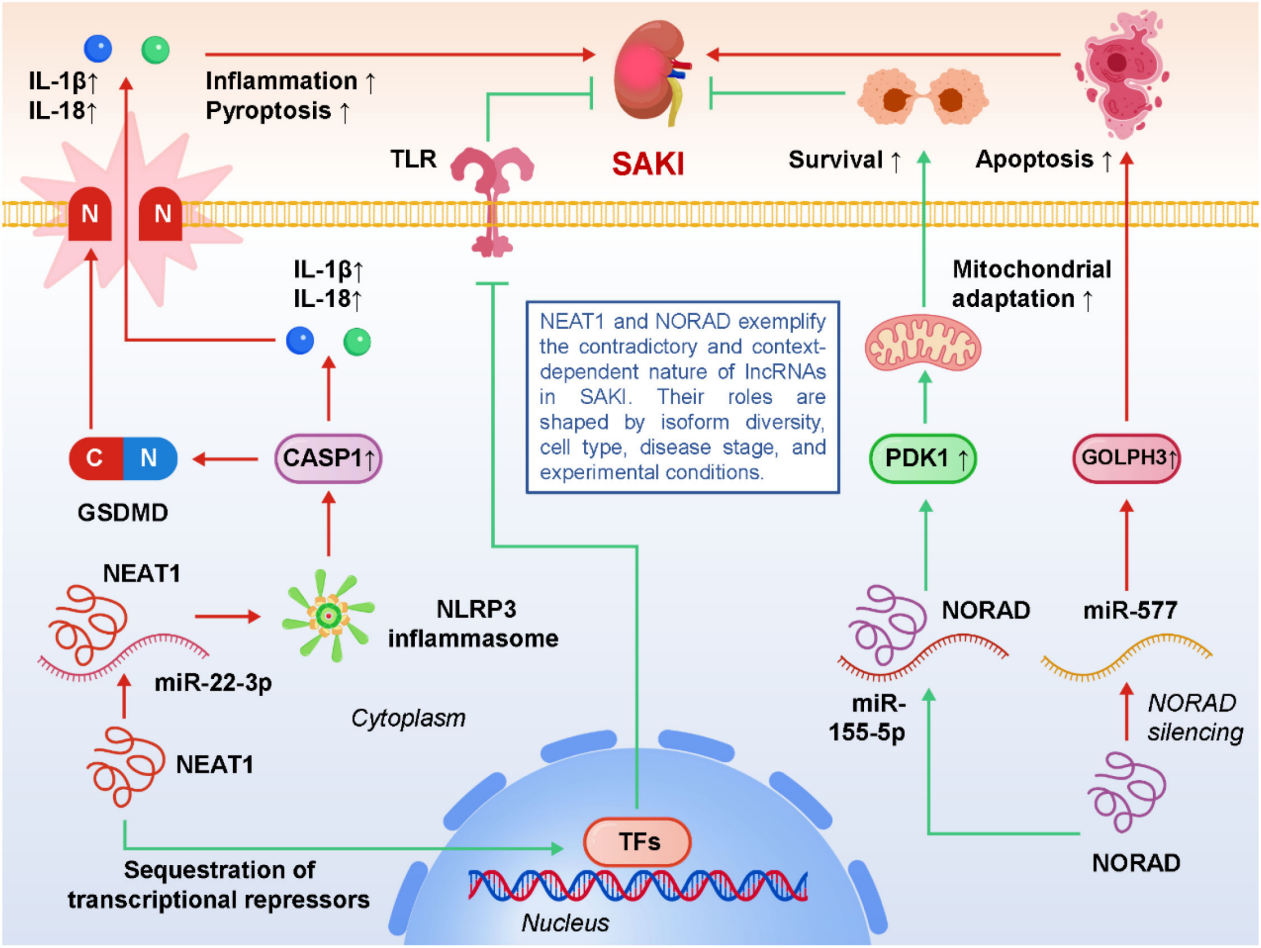
Contradictory and context-dependent roles of the lncRNAs nuclear enriched abundant transcript 1 (NEAT1) and non-coding RNA activated by DNA damage (NORAD) in sepsis-associated acute kidney injury (SA-AKI).

Notably, some lncRNAs have demonstrated seemingly contradictory functions in the context of SA-AKI, underscoring the complexity, plasticity, and context-dependence of non-coding RNA biology (89–91). A prominent example is NEAT1, a nuclear-enriched transcript involved in paraspeckle assembly and stress regulation. Several studies have reported that NEAT1 aggravates renal injury by sponging miR-22-3p and activating the NLRP3 inflammasome, thereby promoting pyroptosis and the release of IL-1β and IL-18, which intensify inflammation and tubular cell death (92). In sharp contrast, other reports suggest that NEAT1 upregulation may mitigate LPS-induced injury by stabilizing nuclear paraspeckles, sequestering transcriptional repressors, and buffering stress-responsive gene expression, ultimately conferring protection. These discrepancies may be attributed to multiple layers of complexity: differences in NEAT1 isoforms (NEAT1_1 vs NEAT1_2) with distinct structural and functional roles, variability in experimental models (immortalized HK-2 cells, primary tubular epithelial cells, CLP mice, or endotoxemia models), and importantly, the temporal dynamics of sepsis, since lncRNA expression often fluctuates rapidly between early hyperinflammation and later immunosuppressive phases. A similar paradox is observed with NORAD, a cytoplasmic lncRNA best known for maintaining genomic stability under stress conditions (93). On the one hand, NORAD has been shown to protect proximal tubular cells from dysfunction by regulating the miR-155-5p/PDK1 axis, thereby supporting pro-survival signaling and metabolic adaptation. On the other hand, experimental silencing of NORAD in different septic models attenuated renal inflammation and apoptosis via the miR-577/GOLPH3 pathway, suggesting that its biological role may flip depending on cellular stress intensity, dominant signaling cascades, or specific microenvironmental cues (94). This duality implies that NORAD may not have a fixed “protective” or “pathogenic” identity, but instead acts as a molecular rheostat whose functional output depends on the broader regulatory landscape. Beyond NEAT1 and NORAD, such discrepancies likely extend to other lncRNAs as well, reflecting the heterogeneity of sepsis itself. Factors such as species differences (mouse vs rat vs human), experimental induction methods (LPS injection vs CLP vs cecal slurry), cell type–specific heterogeneity (tubular epithelial cells, podocytes, endothelial cells, infiltrating macrophages), and technical variations in RNA detection (qPCR vs RNA-seq, bulk vs single-cell approaches) all influence observed outcomes. In addition, many studies measure lncRNA expression at single time points, which may fail to capture dynamic shifts in lncRNA activity across different phases of sepsis progression (95, 96). These inconsistencies highlight a critical lesson: lncRNAs should not be simplistically categorized as “protective” or “pathogenic.” Instead, they operate within context-specific regulatory networks that are shaped by stimulus type, cell lineage, isoform usage, and disease stage. Resolving these contradictions will require rigorous validation across complementary models (LPS vs CLP), use of both *in vitro* and *in vivo* systems, isoform-specific functional dissection, and ideally correlation with clinical samples from septic patients. Such efforts will be essential to establish the true functional landscape of lncRNAs in SA-AKI, enabling precise identification of therapeutic targets and avoiding misinterpretation of context-dependent effects. **Figure 6** depicts how NEAT1 and NORAD act as context-dependent molecular rheostats in SA-AKI, displaying either protective or pathogenic roles depending on isoforms, cell type, disease phase, and experimental setting.

## 4 Therapeutic implications and opportunities

The expanding recognition of lncRNAs as central regulators of SA-AKI raises the possibility of leveraging them as therapeutic targets or biomarkers. To enhance translational relevance, we summarize experimentally validated and clinically observed lncRNAs associated with SA-AKI in **Table 1**, highlighting their pathogenic or protective roles, underlying mechanisms, and sample sources.

**Table 1:** Experimentally and clinically relevant lncRNAs associated with sepsis-associated acute kidney injury (SA-AKI).

lncRNA	Type	Mechanism/target pathway	Sample type	Reference
PVT1	Pathogenic	Activates NF-κB signaling and NLRP3 inflammasome via miR-20a-5p/NLRP3 and miR-17-5p/NF-κB axes, promoting inflammation and pyroptosis	Renal tissue, tubular epithelial cells	Deng et al., 2021 (18); Yuan et al. (55)
MEG3	Pathogenic	Promotes pyroptosis by regulating the miR-18a-3p/GSDMD pathway	Renal tubular epithelial cells	Deng et al., 2021 (20)
MALAT1	Pathogenic	Induces ferroptosis by interacting with FUS to stabilize ACSF2 mRNA, enhancing lipid peroxidation and oxidative stress	Renal tissue, tubular epithelial cells	Duan et al. (59)
KCNQ1OT1	Pathogenic	Activates p38/MAPK-mediated NF-κB signaling through the miR-212-3p/MAPK1 axis	Renal tissue	Wang et al., 2021 (47)
CRNDE	Pathogenic	Enhances renal inflammation via the TLR3/NF-κB signaling pathway	Renal tissue	Sun et al. (52)
GAS6-AS2	Pathogenic	Promotes oxidative stress by regulating the miR-136-5p/OXSR1 axis	Renal tissue, tubular epithelial cells	Cui et al. (60)
CASC2	Protective	Suppresses NF-κB activation and inflammation via miR-155 or miR-545-3p/PPARA axis	Renal tissue	Wang et al., 2020 (53); Hu et al. (21)
GAS5	Protective	Inhibits pyroptosis through activation of the SIRT1/PGC-1α/Nrf2 antioxidant pathway	Renal tissue, tubular epithelial cells	Ling et al. (22)
XIST	Protective	Attenuates inflammation and apoptosis via the miR-155-5p/WWC1 axis	Renal tissue	Wang & Cao (23)
LINC00261	Protective	Restrains NF-κB signaling through the miR-654-5p/SOCS3 axis	Renal tissue	Li et al. (54)
MIAT	Context-dependent	Regulates autophagy by stabilizing BECN1 mRNA via PTBP1 interaction	Renal tubular epithelial cells	Xu et al., 2023 (74)
SNHG14	Context-dependent	Modulates autophagy through miR-373-3p/ATG7 and miR-495-3p/HIPK1 pathways	Renal tissue	Yang et al. (75), Yang et al, (76)
NEAT1	Context-dependent	Either promotes NLRP3 inflammasome activation (miR-93-5p/TXNIP) or confers stress adaptation depending on isoform and context	Renal tissue, tubular epithelial cells	Yang et al., 2021 (34); Xue et al. (48)
NORAD	Context-dependent	Regulates apoptosis and metabolic stress via miR-155-5p/PDK1 or miR-577/GOLPH3 axes	Renal tissue	Lu et al.,2024 (93), Xie et al,. 2022 (94)

### 4.1 Pharmacological agents targeting lncRNAs.

Several small molecules and natural products have demonstrated renoprotective effects in experimental models of SA-AKI by modulating lncRNA expression, suggesting that conventional pharmacological agents can be repositioned to indirectly target non-coding RNA networks. Resveratrol, a polyphenolic compound with well-characterized antioxidant and anti-inflammatory properties, has been shown to alleviate sepsis-induced kidney injury by suppressing the MALAT1/miR-205 axis. By reducing MALAT1 expression, resveratrol enhances miR-205 availability, leading to inhibition of NF-κB activation, decreased pro-inflammatory cytokine release, and attenuation of tubular apoptosis (97). These findings position resveratrol not only as a metabolic and vascular protector but also as a potential modulator of lncRNA-driven inflammatory networks. Similarly, curcumin, the bioactive component of turmeric long known for its immunomodulatory effects, mitigates septic renal injury by downregulating PVT1, a lncRNA consistently implicated in pro-inflammatory signaling. Suppression of PVT1 dampens NF-κB activation and NLRP3 inflammasome priming, thereby reducing renal inflammation and tubular cell pyroptosis (18, 19). Preclinical animal studies revealed that curcumin administration significantly lowered serum creatinine and blood urea nitrogen levels, ameliorated histological evidence of tubular necrosis, and improved survival, underscoring its therapeutic promise. Importantly, these effects occurred in parallel with PVT1 inhibition, raising the possibility that curcumin may exert part of its renoprotective activity through lncRNA modulation rather than purely through classical antioxidant pathways. In addition, paclitaxel, beyond its established role as a microtubule-stabilizing chemotherapeutic agent, has been reported to protect against SA-AKI by downregulating MALAT1 and disrupting the miR-370-3p/HMGB1 axis. This intervention reduces HMGB1 release, a critical mediator of sepsis-induced systemic inflammation and tubular injury, thereby limiting the inflammatory cascade and preserving renal function (17). The paclitaxel example is particularly intriguing because it highlights how drugs originally developed for non-renal indications may be repurposed to modulate lncRNA signaling in sepsis. Collectively, these examples highlight the possibility of repurposing widely studied bioactive compounds to modulate lncRNA-dependent pathways, offering a more immediate translational route compared with *de novo* RNA therapeutics that face delivery and stability challenges. Moreover, small molecules and natural products may synergize with direct RNA-based interventions, serving as adjunctive therapies to fine-tune lncRNA expression *in vivo*. Nevertheless, their pleiotropic actions also raise challenges (98, 99). Because resveratrol, curcumin, and paclitaxel influence numerous molecular targets, it remains difficult to attribute their renoprotective effects exclusively to lncRNA modulation. Off-target effects, dose-dependent toxicity, and variability in bioavailability further complicate mechanistic interpretation. Therefore, rigorous mechanistic studies are required to dissect the causal relationship between small-molecule treatment and lncRNA regulation. This will involve combining RNA interference or CRISPR-based lncRNA knockdown with pharmacological treatment in septic models to confirm whether specific lncRNA pathways are necessary for therapeutic efficacy. Furthermore, clinical validation in well-designed cohorts is essential to determine whether changes in circulating or urinary lncRNA profiles accompany clinical improvement in patients receiving these compounds. Such studies would provide stronger evidence that natural products and small molecules can indeed be repurposed as lncRNA-targeted therapies in sepsis-associated AKI.

### 4.2 RNA-based therapeutics

Direct targeting of lncRNAs represents a particularly attractive therapeutic strategy in SA-AKI, given their central position in orchestrating inflammation, oxidative stress, and multiple forms of programmed cell death (100–102). Unlike conventional small molecules that often act on protein targets, RNA-based interventions offer the advantage of modulating disease at the transcriptional or post-transcriptional level, where lncRNAs exert their regulatory effects. Antisense oligonucleotides (ASOs) and small interfering RNAs (siRNAs) are the most widely explored platforms, capable of selectively silencing pathogenic lncRNAs such as PVT1 or NEAT1. By reducing transcript abundance, these approaches can suppress NF-κB activation, limit NLRP3 inflammasome priming, and attenuate oxidative stress pathways, thereby alleviating tubular injury (34, 55, 103). Conversely, lncRNA mimics or viral vector–based overexpression systems can be deployed to restore beneficial transcripts such as GAS5, which reduces pyroptosis through the SIRT1/PGC-1α/Nrf2 axis, or CASC2, which inhibits NF-κB activation, highlighting the potential of lncRNA augmentation as a cytoprotective therapy (53). Recent advances in RNA chemistry have substantially improved the feasibility of these approaches. Modifications such as phosphorothioate backbones, 2′-O-methyl ribose substitutions, or locked nucleic acid (LNA) analogs increase nuclease resistance, enhance stability in circulation, and reduce immunogenicity (104–106). These innovations minimize off-target binding and allow for sustained activity *in vivo*, addressing long-standing barriers to clinical application. Equally important are breakthroughs in RNA delivery platforms. Lipid nanoparticles (LNPs), validated in mRNA vaccine technology, provide efficient cytoplasmic delivery of siRNAs and ASOs. GalNAc conjugates facilitate hepatocyte-specific uptake and may be adapted for kidney-targeted delivery by leveraging renal receptor biology. Engineered exosomes, derived from immune or stem cells, represent another promising vehicle, as they can naturally home to injured tissues and be loaded with therapeutic RNA cargo (107–109). Early reports suggest that exosomes modified with kidney-homing peptides or antibodies could achieve greater nephron specificity, an exciting frontier for SA-AKI therapy. While proof-of-concept studies in rodent sepsis models demonstrate encouraging efficacy, translation to the clinic remains challenging. One major hurdle is rapid renal clearance of naked RNA molecules, which limits bioavailability and necessitates frequent dosing. Another is the heterogeneous uptake across nephron segments: proximal tubular cells, podocytes, and endothelial cells may respond differently to RNA delivery, complicating uniform therapeutic effects. In addition, lncRNAs function within intricate ceRNA and protein interaction networks; silencing or overexpressing a single lncRNA may inadvertently perturb broader regulatory axes, leading to unintended consequences. Off-target gene modulation and immune activation remain concerns, particularly in the inflammatory milieu of sepsis. Nonetheless, the rapid maturation of RNA-based technologies—as evidenced by their success in genetic disorders, cardiovascular disease, and oncology—provides a strong foundation for lncRNA-targeted therapies in SA-AKI. The first FDA-approved ASO therapies and the clinical validation of siRNA-based drugs demonstrate that RNA modulation is no longer theoretical but clinically actionable (110, 111). Applying these lessons to lncRNAs will require careful optimization of delivery systems, context-specific target selection, and robust validation in large-animal sepsis models and patient-derived samples. Looking ahead, combining lncRNA-based therapeutics with pharmacological agents or supportive interventions such as CRRT may yield synergistic benefits, enabling a precision medicine approach tailored to the molecular drivers of renal injury in sepsis.

### 4.3 Exosome-mediated delivery.

Exosomes, nanoscale extracellular vesicles (30–150 nm) naturally secreted by most cell types, have emerged as highly promising delivery vehicles for RNA-based therapeutics owing to their intrinsic stability, low immunogenicity, and innate capacity for intercellular communication (112–114). Unlike synthetic nanoparticles, exosomes are enriched in lipids, proteins, and nucleic acids that reflect their cell of origin, making them ideal carriers for lncRNAs or miRNAs in disease contexts such as SA-AKI. Their ability to cross biological barriers and protect RNA cargo from degradation in circulation further enhances their translational appeal (115). In the context of SA-AKI, endothelial progenitor cell (EPC)-derived exosomes carrying miR-21-5p have been shown to downregulate RUNX1, thereby attenuating tubular apoptosis, suppressing inflammation, and improving renal function (116). This compelling proof-of-concept demonstrates that exosomal RNA cargo can exert protective effects in sepsis models, validating the therapeutic potential of exosome-based delivery. Importantly, exosomes can be engineered to encapsulate therapeutic lncRNAs or miRNAs and tailored to enhance renal tropism. Techniques such as electroporation, lipid-based transfection, or RNA overexpression in donor cells enable the enrichment of specific RNA species within vesicles (117, 118). Surface modifications, including conjugation with kidney-targeting ligands, antibodies, or peptides, can direct exosomes to tubular epithelial cells, podocytes, or glomerular endothelial cells, thereby maximizing efficacy while minimizing systemic off-target effects. These innovations circumvent some of the limitations associated with synthetic nanoparticles, such as rapid renal clearance, poor biocompatibility, and risk of immune activation. Moreover, exosomes can be derived from autologous sources (e.g., patient-derived mesenchymal stem cells), raising the possibility of personalized therapy that leverages an individual’s own vesicles for RNA delivery. Beyond delivery advantages, exosomes themselves may contribute to paracrine signaling that supports tissue repair (119, 120). For example, mesenchymal stem cell–derived exosomes have been reported to reduce oxidative stress, enhance angiogenesis, and promote mitochondrial transfer, effects that could synergize with therapeutic RNA cargo to restore renal homeostasis. This dual functional capacity—both as a vehicle and as an active modulator of injury responses—distinguishes exosomes from other delivery platforms. Despite their promise, significant hurdles remain before exosome-based therapeutics can be translated into clinical practice. One major barrier is the scalability and standardization of exosome production. Current isolation methods such as ultracentrifugation, size-exclusion chromatography, or precipitation lack uniformity, raising concerns about reproducibility and purity. Additionally, the efficiency and consistency of RNA loading remain variable, with no consensus on the optimal engineering approach. Another unresolved issue is biodistribution: although surface modification can enhance renal tropism, exosomes naturally accumulate in the liver, spleen, and lungs, raising questions about dose efficiency. Moreover, the long-term safety of repeated exosome administration in humans is unclear, particularly regarding unintended immune modulation or horizontal gene transfer. Taken together, pharmacological modulation, RNA-based therapeutics, and exosome-mediated delivery collectively underscore the translational potential of lncRNA-targeted strategies in SA-AKI. Exosomes, in particular, offer a biologically compatible and versatile platform for delivering RNA cargo directly to renal cells, potentially overcoming key limitations of synthetic carriers. However, realizing this potential will require rigorous preclinical studies to optimize production, loading, and targeting strategies, followed by carefully designed clinical trials to evaluate safety, efficacy, and biomarker-guided patient selection. If these challenges can be addressed, exosome-mediated delivery of therapeutic lncRNAs or miRNAs may evolve into a powerful and personalized intervention for mitigating renal injury in sepsis.

Beyond their therapeutic potential, lncRNAs also hold promise as diagnostic and prognostic biomarkers in sepsis-associated acute kidney injury. Emerging evidence suggests that lncRNA expression profiles in renal tissue, circulating blood, or urine may reflect early tubular stress and inflammatory activation before overt changes in serum creatinine become evident. Incorporating lncRNA-based signatures into clinical workflows could therefore assist in patient stratification, identifying septic patients at high risk for AKI development or progression. Importantly, lncRNAs are unlikely to function as standalone biomarkers; instead, multi-marker strategies that integrate lncRNAs with established AKI biomarkers such as KIM-1, NGAL, and TIMP2·IGFBP7 may improve predictive accuracy and temporal resolution. Such combinatorial approaches could enable earlier risk assessment, guide personalized monitoring, and ultimately facilitate more precise and timely interventions in sepsis-associated AKI. From a clinical perspective, the lack of disease-specific biomarkers remains a major barrier to improving the management and outcomes of sepsis-associated acute kidney injury. Currently used biomarkers largely reflect nonspecific tubular stress or functional decline and often fail to provide sufficient sensitivity or specificity when translated into routine clinical practice (121). In this context, lncRNAs involved in SA-AKI pathogenesis may represent a new class of mechanism-based biomarkers that more directly capture disease-driving inflammatory, metabolic, and cell death pathways. Rather than replacing existing markers, lncRNA signatures could complement conventional biomarkers to enable earlier diagnosis, refined risk stratification, and improved prognostic assessment. Such disease-targeted biomarker strategies may ultimately facilitate more timely and personalized clinical decision-making in patients with sepsis-associated acute kidney injury.

## 5 Challenges and future directions

Recent advancements in high-resolution transcriptomic technologies, particularly single-cell RNA sequencing (scRNA-seq) and spatial transcriptomics, are poised to revolutionize our understanding of complex diseases like SA-AKI (122, 123). These technologies offer unprecedented resolution to explore the heterogeneity of kidney cell populations and to map lncRNA expression in specific renal compartments, such as tubular cells, podocytes, and immune cells. By enabling the study of spatially resolved gene expression in tissue samples, they have the potential to uncover previously hidden molecular pathways that govern injury responses and repair mechanisms in AKI. Despite these exciting prospects, there are still several challenges. For instance, scRNA-seq often struggles with the detection of low-abundance lncRNAs, and isoform-specific resolution remains limited. Furthermore, most studies using these technologies still focus on a single time point, limiting the understanding of temporal dynamics in sepsis progression (124, 125). Future research integrating single-cell, spatial, and epitranscriptomic profiling, alongside functional validation and clinical correlation, will be crucial to fully harness these technologies for translating molecular insights into actionable biomarkers and therapeutic targets for SA-AKI. Despite compelling preclinical evidence, the translation of lncRNA research in SA-AKI from bench to bedside remains nascent, constrained by model fidelity, biological heterogeneity, and delivery barriers. At present, the vast majority of functional studies rely on LPS-stimulated tubular cells or rodent cecal ligation and puncture (CLP) models, which capture certain aspects of sepsis but fall short of reflecting the full spectrum of human disease (126, 127). Human sepsis is clinically heterogeneous, spanning hyperinflammatory and immunosuppressive endotypes, often in the setting of comorbidities such as diabetes, hypertension, or chronic kidney disease that are absent in young healthy rodents. Furthermore, rodent kidneys differ from human kidneys in nephron density, immune cell composition, and metabolic profiles, limiting direct extrapolation. To improve model fidelity, it will be important to harmonize polymicrobial sepsis, endotoxemia, and ischemia–reperfusion models and to incorporate large-animal or humanized systems, which better recapitulate hemodynamics and immune–metabolic complexity. In parallel, emerging kidney organoids and kidney-on-chip platforms provide reductionist yet physiologically relevant models to study lncRNA–miRNA–protein networks under controlled microenvironmental conditions, bridging the gap between rodent work and human pathology (128, 129). Functional heterogeneity represents another formidable obstacle. The same lncRNA may switch from protective to pathogenic depending on isoform usage, subcellular localization (e.g., nuclear paraspeckles vs cytoplasmic ceRNA pools), cell lineage (proximal tubule, glomerular podocyte, endothelial cell, or infiltrating immune cells), stimulus intensity, and temporal stage of sepsis. This explains why transcripts such as NEAT1 and NORAD have yielded contradictory results across studies. Resolving such complexity will require cutting-edge technologies: single-cell and spatial transcriptomics to map lncRNA dynamics across nephron segments; isoform-resolved long-read sequencing to discriminate overlapping transcripts; RNA–protein interactome mapping (RIP/CLIP) to identify functional partners; and epitranscriptomic profiling (m^6^A, RNA editing) to capture modifications that influence lncRNA stability and function. Functional dissection can be advanced by CRISPRi/a tiling screens, perturb-seq, and domain-specific mutagenesis, which together enable causal assignment of lncRNA effects in a cell-type–specific manner. On the therapeutic front, direct RNA interventions such as ASOs, siRNAs, and lncRNA mimics face technical bottlenecks. Naked RNA molecules suffer from rapid renal clearance, poor stability, and endosomal trapping, while off-target ceRNA competition raises the risk of unintended gene modulation (107, 130). Overcoming these hurdles will depend on advances in kidney-tropic delivery systems. Strategies include receptor-guided ligands for proximal tubule uptake (e.g., megalin- or cubilin-targeting moieties), optimized lipid nanoparticles with renal tropism, or engineered exosomes decorated with kidney-homing peptides. Rigorous biodistribution and PK–PD studies, coupled with target engagement readouts such as lncRNA knockdown efficiency, miRNA de-repression, or changes in NF-κB/NLRP3 activity, will be essential benchmarks for clinical readiness. Moreover, because lncRNAs intersect with host defense, precise dose–timing strategies must be established to avoid compromising antimicrobial immunity (131, 132). In this context, combination regimens—integrating lncRNA-targeted interventions with hemodynamic optimization, anti-inflammatory drugs, or antioxidant therapies—are likely to prove superior to monotherapy. For clinical translation, the field also requires fit-for-purpose biomarkers. Circulating or urinary lncRNAs could complement established AKI markers such as KIM-1, NGAL, or TIMP2·IGFBP7, improving early detection, risk stratification, and monitoring of treatment response (133–135). Multi-marker panels that combine lncRNAs with protein biomarkers and clinical phenotypes may enable enrichment strategies for clinical trials and facilitate adaptive platform trial designs, accelerating the evaluation of candidate therapies. Finally, achieving impact will demand field-level rigor and standardization. This includes pre-registered experimental protocols, standardized endpoints (renal histology, function, survival), stratification by sex, age, and comorbidities, and transparent data sharing. Such measures will be critical for reconciling contradictory findings (e.g., NEAT1, NORAD), reducing the risk of publication bias, and ensuring reproducibility. By systematically addressing these challenges, the field can progress toward unlocking the full translational potential of lncRNAs—transforming them from experimental curiosities into actionable biomarkers and therapeutic nodes for improving outcomes in sepsis-associated AKI.

An additional layer of biological heterogeneity that warrants further investigation is the influence of sex and age on lncRNA expression and function in sepsis-associated acute kidney injury. Accumulating evidence from related inflammatory and renal disease models suggests that sex hormones, age-associated epigenetic remodeling, and immunometabolic differences can shape lncRNA transcriptional programs and downstream stress responses. These factors may partially explain the observed variability in SA-AKI susceptibility, severity, and recovery between male and female patients, as well as across different age groups. However, most existing studies on lncRNAs in SA-AKI do not stratify analyses by sex or age, limiting the interpretability and translational relevance of current findings. Future investigations integrating sex- and age-stratified designs, ideally combined with single-cell or spatial transcriptomic approaches, will be essential to delineate context-dependent lncRNA networks and to improve precision risk assessment and therapeutic targeting in SA-AKI.

## 6 Conclusion

Long non-coding RNAs (lncRNAs) have emerged as pivotal regulators in the pathogenesis of sepsis-associated acute kidney injury (SA-AKI), functioning as a double-edged sword that can either exacerbate or mitigate renal injury depending on the molecular and cellular context. Acting through diverse mechanisms, lncRNAs orchestrate key pathogenic processes including inflammation, pyroptosis, ferroptosis, autophagy, and apoptosis, thereby shaping the balance between renal damage and repair (136, 137). On one hand, pathogenic lncRNAs such as PVT1, MEG3, and MALAT1 amplify NF-κB activation, inflammasome signaling, mitochondrial dysfunction, or oxidative stress, ultimately aggravating tubular and endothelial injury. On the other, protective transcripts like CASC2, GAS5, XIST, and LINC00261 restrain excessive inflammation, preserve mitochondrial homeostasis, suppress cell death programs, and facilitate stress adaptation, underscoring the plasticity of lncRNA-mediated regulation. This duality highlights both the complexity of lncRNA biology and their promise as diagnostic biomarkers and therapeutic targets. Circulating and urinary lncRNAs have shown potential as early, non-invasive indicators of renal stress, offering higher sensitivity than conventional biomarkers such as serum creatinine, which often lag behind actual injury (138, 139). Furthermore, therapeutic modulation of lncRNA networks is beginning to demonstrate feasibility: small molecules like resveratrol, curcumin, and paclitaxel can downregulate pathogenic lncRNAs and attenuate inflammation, while antisense oligonucleotides, siRNAs, or viral vectors can silence deleterious transcripts (PVT1, NEAT1) or restore protective ones (GAS5, CASC2). In addition, engineered exosomes and nanoparticle systems provide emerging delivery platforms for tissue-specific lncRNA modulation, raising the possibility of precision medicine tailored to renal cellular targets.

Nevertheless, substantial challenges must be addressed before clinical translation can be realized. First, the functional heterogeneity of lncRNAs remains a major barrier: the same molecule may exhibit opposite effects in different models, as seen with NEAT1 and NORAD, reflecting isoform diversity, temporal dynamics, and cell-type specificity. Second, mechanistic dimensions such as ferroptosis, immunometabolic reprogramming, and organ cross-talk (e.g., the gut–kidney axis) remain underexplored, limiting a holistic understanding of lncRNA-mediated injury networks. Third, therapeutic delivery is still a bottleneck: while chemical modifications have improved RNA stability, challenges such as rapid renal clearance, off-target interactions, and immune activation must be overcome. Finally, most current evidence derives from rodent sepsis models, and robust validation in large-animal studies and prospective human cohorts is urgently needed to establish clinical relevance. Looking ahead, integrating multi-omics approaches (transcriptomics, epitranscriptomics, single-cell sequencing) with advanced functional genomics tools (CRISPRi/a, perturb-seq, RBP interactome mapping) will be crucial for delineating context-specific lncRNA functions and identifying tractable targets. In parallel, clinical research should focus on validating lncRNAs as biomarkers for early detection, prognosis, and therapeutic response monitoring. Importantly, lncRNA-based interventions may be most effective when deployed in combination with established strategies such as hemodynamic stabilization, anti-inflammatory agents, or antioxidant therapies, reflecting the multifactorial nature of SA-AKI.

In summary, lncRNAs occupy a central and multifaceted role in SA-AKI, bridging inflammation, oxidative stress, and programmed cell death pathways. While their dual roles underscore biological complexity and translational challenges, they also provide unprecedented opportunities for innovation in diagnostics and therapeutics. With continued mechanistic exploration, technological advances in delivery systems, and rigorous clinical validation, the integration of lncRNA biology into the sepsis field has the potential to transform the management of AKI and improve outcomes for critically ill patients.
